# Taohong Siwu Decoction Promotes Osteo-Angiogenesis in Fractures by Regulating the HIF-1*α* Signaling Pathway

**DOI:** 10.1155/2022/6777447

**Published:** 2022-09-20

**Authors:** Zhi Tang, Ming Yin, Yuxing Guo, Wei Li, Fei Sun, Yonglin Guo, Zhenzhong Chen, Biao Zhou

**Affiliations:** ^1^Department of Orthopedics, Xiangtan Chinese Medicine Hospital, Xiangtan, Hunan, China; ^2^Department of Orthopedics, Hunan University of Chinese Medicine, Changsha, Hunan, China; ^3^Department of Orthopedics, Hunan Academy of Traditional Chinese Medicine Affiliated Hospital, Changsha, Hunan 410208, China; ^4^Department of Orthopedics, Xiangtan Hospital Affiliated to Nanhua University, Xiangtan, Hunan, China; ^5^Department of Orthopedics, Wangjing Hospital of Chinese Academy of Chinese Medical Science, Beijing, China

## Abstract

**Background:**

Vascular damage is a major consequence of bone fracture. Taohong Siwu decoction (TSD) can raise the expression of vascular endothelial growth factor (VEGF) in fracture healing. However, its molecular mechanism in promoting angiogenesis is still unknown. The aim of this study was to investigate the potential mechanisms of TSD in the regulation of osteo-angiogenesis in fracture healing.

**Methods:**

A rat tibial fracture model was established. After low- (4.5 g·kg^−1^), medium- (9 g·kg^−1^), and high-dose TSD (18 g·kg^−1^) and panax notoginsenoside (25 mg kg^−1^) treatment, hematoxylin-eosin staining was employed to visualize pathological changes in bone tissues. The levels of cytokines (interleukin (IL)-2, tumor necrosis factor-*α* (TNF-*α*), IL-6, and IL-1*β*), thromboxane B2 (TXB2), and 6 ketone prostaglandin F1*α* (6-Keto-PGF1*α*) were quantified by enzyme-linked immunosorbent assay (ELISA). Immunofluorescence was used to identify the rat aortic endothelial cells (RAECs). Control serum, 10% TSD-containing serum, and 10% TSD-containing serum combined with hypoxia-inducible factor-1*α* (HIF-1*α*) inhibitor were used to treat the RAECs and rat osteoblasts. Transwell migration assay was utilized to examine the migration of the RAECs. The Matrigel tubulogenesis assay was used for the assessment of angiogenesis. The expression of angiogenesis- (von Hippel-Lindau tumor suppressor (VHL), HIF-1*α*, VEGF, angiopoietin-2 (Ang-2), and pVHL) and osteogenesis-related (alkaline phosphatase (ALP), runt-related transcription factor 2 (Runx2), and osteopontin-1 (OPN-1)) protein and gene was detected by western blot and quantitative real-time PCR (qRT-PCR).

**Results:**

Compared with the model group, TSD increased the trabecular bone areas, numbers, and thicknesses in fractured rats. In the plasma, the levels of cytokines and TXB2 in the middle- and high-dose TSD group were significantly lower than those in the model group (*P* < 0.01). The 6-keto-PGF1*α* content was increased by middle- and high-dose TSD intervention (*P* < 0.01). Compared to the control serum group, the angiogenesis and migration of the RAECs were enhanced in the TSD group (*P* < 0.001). The expression of HIF-1*α*, VEGF, and Ang-2 in the TSD group upregulated significantly (*P* < 0.001). VHL and pVHL were inhibited under TSD-containing serum treatment (*P* < 0.001). ALP, Runx2, and OPN-1 were increased obviously in the TSD group (*P* < 0.001). Nevertheless, the HIF-1*α* inhibitor reversed these changes (*P* < 0.001).

**Conclusion:**

TSD promotes angiogenesis and osteogenesis by regulating the HIF-1*α* signaling pathway. Meanwhile, it can effectively reduce the risk of inflammation and improve blood circulation.

## 1. Introduction

Bone fracture is a clinically common orthopedic disease, which refers to the destruction of the bone or trabecular bone [1]. Approximately 10% of fractures fail to heal normally, which results in pain, disability, and repetitive operative interventions [2]. The process of fracture treatment is affected by multiple biological factors, including inflammation [3], oxygen content [4], hormone [5], and mechanical stimulation [6]. It is necessary to explore effective agents or methods to repair the condition of the injury in fracture healing.

Chinese herbal medicines (CHMs) have the merits of high cost effectiveness, few side effects, and suitability for long-term use. They are widely used to treat bone-related diseases, such as osteoporosis and fracture [7–9]. Taohong Siwu decoction (TSD) is a formula consisting of six traditional CHMs of *Semen Persicae*, *Flos Carthami*, *Angelica Sinensis*, *Radix Paeoniae Alba*, *Rhizoma Chuanxiong*, and *Radix Rehmanniae Praeparata* [10]. TSD was demonstrated to maintain the effects of blood activation [10], pain relief, and anti-inflammation [11]. It is often utilized in gynecological and cardiovascular diseases [12, 13]. Accumulative evidence suggests that TSD may exert therapeutic effects on bone injury healing by promoting bone remodeling [14, 15]. TSD may contribute to blood circulation and remove blood stasis, but its exact molecular mechanism needs further exploration.

The fracture causes vascular injury, which leads to the decrease or even interruption of blood supply at the fracture site. Due to the vascular injury, the fracture site becomes hypoxic [16], thus triggering the hypoxia inducible factor (HIF) pathway and upregulating the expression of hypoxia-inducible factor-1*α* (HIF-1*α*). Under hypoxia conditions, HIF-1*α* accumulates in endothelial cells and binds to the vascular permeability factor (VEGF) gene promoter to induce VEGF gene expression [17]. Angiogenesis is a pivotal process in fracture repair, and VEGF is a key regulator of angiogenesis. Besides, VEGF is the basic medium of osteogenic reaction in osteogenesis [18]. Our preliminary experiments indicate that TSD can promote the expression of VEGF, but the mechanism of intervention on the HIF-1*α* signaling pathway upstream of VEGF has not been clear yet [19]. In this study, we will investigate the effect and molecular mechanism of TSD on early angiogenesis of fractures by observing the expression of angiogenesis-related proteins and cytokines in early fracture of rats treated with TSD. This study may provide new ideas and methods for the early treatment of fracture by TSD.

## 2. Materials and Methods

### 2.1. Animals

In this experiment, Wistar rats (weighing 200–250 g) were purchased from the Hunan University of Traditional Chinese Medicine. We established the tibial fracture model in rats on the basis of previous methods [20, 21]. In brief, rats were anesthetized, and an approximately 8 mm longitudinal incision was made under the right knee joint. After separating the muscle fascia, the tibia was cut at the upper middle third of the tibia, and the surgical incision was closed. After modeling, rats were administered daily by gavage with appropriate doses of TSD or saline. The experimental drugs, including TSD and panax notoginsenoside (PNS), were provided by the Hunan University of Traditional Chinese Medicine. Rats were randomly divided into five groups: a sham group, a model group, a low-dose TSD group (4.5 g·kg^−1^), a medium-dose TSD group (9 g·kg^−1^), a high-dose TSD group (18 g·kg^−1^), and positive control (25 mg kg^−1^ PNS treatment) group, with six rats in each group. The preparation of TSD was followed as previously reported [22]. The dose of each rat by gavage was calculated according to the equivalent adult dose of the body surface area. Following 28 days of continuous administration of drug or normal saline, the rats were sacrificed, and blood samples and bone tissues were collected. Ethylene diamine tetraacetic acid (EDTA, E9884, Sigma, USA) or heparin (AWH0144a, Abiowell, China) were used as anticoagulants. This study was approved by the experimental animal welfare and ethics committee of Hunan University of Traditional Chinese Medicine, and all experiments were conducted in accordance with the guidelines formulated by the committee.

### 2.2. Preparation of Drug-Containing Serum

The drug-containing serum was prepared as we described previously [22]. In brief, TSD was gavaged to rats continuously twice daily for one week, as mentioned above. For the sham group, equal amounts of saline were given. One week after drug treatment, blood was collected from the abdominal aorta of the rats. The serum containing the drug was obtained after centrifugation.

### 2.3. Isolation and Characterization of Rat Aortic Endothelial Cells (RAECs)

The aorta was isolated by thoracotomy from the rats, and the endothelial cells were isolated according to the previous literature [23]. Cell slides were washed three times with phosphate buffer saline (PBS) after removal and then fixed with 4% paraformaldehyde (N1012, SolelyBio.mall, China) for 30 min. Cell slides were added with 0.3% Triton X-100 (AWH0299a, Abiowell, China) and permeated at 37°C for 30 min. Samples were rinsed with PBS for 3 min (3 times) and then sealed for 60 min. The appropriate dilution of primary antibody (CD31, 1 : 100, ab222783, Abcam, UK) was dripped into the cell slides and cultured overnight at 4°C. Cell slides were added with 50–100 ul of goat anti-rabbit IgG-labeled fluorescent antibody (1 : 100, SA00013-4, Proteintech, USA) and incubated at 37°C for 90 min. Then, the slides were stained with DAPI working solution for 10 min at 37°C and sealed with buffered glycerol.

### 2.4. Cell Culture

The rat osteoblast cells (CP-R091, Pricella, China) were cultured in a special culture medium (CM-R091, Pricella, China). The RAECs were cultured in DMEM (D5796, Gibco, USA) containing 10% fetal bovine serum (FBS, 10099141, Gibco, USA), 100 U/ml penicillin, and 100 g/ml streptomycin (SV30010, Beyotime Biotechnology, China). CAY10585 is an HIF-1*α* inhibitor (ab144422, Abcam, USA). The cells were assigned randomly to four groups: a control group, a control serum group, a 10% TSD-containing serum group (TSD), and a 10% TSD-containing serum + CAY10585 group (TSD + HIF-1*α* inhibitor). The cells were treated with normal serum, 10% TSD-containing serum, and 10% TSD-containing serum in association with 20 *μ*M CAY10585 for 24 h.

### 2.5. Hematoxylin-Eosin (HE) Staining

Tissue or cellular crawls were fixed with 4% paraformaldehyde for 20 min. Hematoxylin stain (AWI0009a, Abiowell, China) was used to perform nuclear staining for 12–15 min. Eosin staining was performed by immersion for 5 min. The dried cell crawls were sealed with neutral gum. The cell morphology was observed microscopically and photographed.

### 2.6. Methyl Tetrazolium (MTT) Assay

Cells of different groups were digested and inoculated in 96-well plates at a density of 1 × 104 cells/well, 100 *μ*L per well. MTT assay was performed as previously described [24]. In brief, after the cells were treated as above, 10 *μ*L/well of 5 mg/ml MTT (M2128, Sigma, USA) was added to each well, and the cells were incubated at 37°C with 5% CO_2_ for 4 h. The supernatant was discarded by centrifugation, and 150 *μ*L/well of dimethyl sulfoxide (30072418, VETEC, China) was added. The absorbance (OD) value at 490 nm was analyzed by using a microplate reader (MB530, Huisong Technology, China).

### 2.7. Matrigel Tubulogenesis Assay

Matrigel tubulogenesis assay was performed as previously described [25, 26]. In brief, the RAECs were processed with corresponding treatment and inoculated in matrigel (growth factor reduced, 356231, Corning, USA)-coated 96-well plates containing 1.5 × 104 cells per well. The cells were incubated at 37°C for 6 h and photographed under a microscope with five fields of view per well. The images were used to calculate the lumen length using Image-Pro Plus (IPP) 7.1 software.

### 2.8. Transwell Migration Assay

Cells were seeded in the upper layer of a transwell chamber (8 *μ*m, 3428, Corning, USA) in a 6-well plate, with 1 × 105 cells per well. The lower layer of the chambers was filled with a 500 ul complete medium with 10% FBS. After 24 h incubation at 37°C, the cells were fixed with 4% paraformaldehyde for 20 min. 0.1% crystal violet (G1062, Solarbio, China) was stained for 5 min, and the cells were observed under a microscope (DSZ2000X, Beijing Zhongxian Hengye Instrument, China) and photographed. The chambers were decolorized by 10% acetic acid, and the absorbance at OD550 nm was measured by using a microplate reader.

### 2.9. RNA Extraction and Quantitative Real-Time PCR (qRT-PCR)

Isolation of total RNA was performed from RAECs using TRIzol (15596026, Thermo Fisher Scientific Inc, USA), and then, the cDNA was reverse transcribed. qRT-PCR was performed to measure mRNA levels relative to GAPDH expression. The qRT-PCR conditions were as follows: 95°C for 3 min, followed by 40 cycles 95°C for 15 s, 55–60°C for 30 s, 72°C for 1 min, then 72°C for 10 min. The expression of GAPDH was used for standardization, and target messenger RNA (mRNA) expression was quantified by the 2^−ΔΔCt^ method. The required primer sequences are shown in Supplementary Table 1 (Sangon Biotech, Shanghai, China).

### 2.10. Western Blot Analysis

Samples were lysed with 1 × Radio-Immunoprecipitation Assay (RIPA) lysis solution (P0013B, Beyotime Biotechnology, China) containing protease inhibitors (583794, Centihold, China). The lysate was separated on a 9% SDS-PAGE gel and transferred to a polyvinylidene fluoride membrane (EMD Millipore, USA). The membrane was sealed with 5% skimmed milk (P1622, Beijing Prily Gene Technology Co., Ltd., China) and incubated overnight at 4°C with primary antibodies. Then, the membrane was incubated with horseradish peroxidase (HRP) conjugated secondary antibody for 2 h at room temperature. The bands were verified with chemiluminescent reagents. (Thermo Fisher Scientific Inc.). Whole gel image analysis (Vilber, Ltd.) was performed for the OD value measurement. Antibody information is described in Supplementary Table 2.

### 2.11. Enzyme-Linked Immunosorbent Assay (ELISA)

Following sample collection, the samples were centrifuged at 1000*g* for 15 min at 2–8°C, and the supernatant was immediately assayed. The levels of Interleukin (IL) -2 (CSB-E04628r), tumor necrosis factor (TNF)-*α* (CSB-E11987r), IL-6 (CSB-E04640r), IL-1*β* (CSB-E08055r), thromboxane B2 (TXB2, CSB-E08047r), and 6 ketone prostaglandin F1*α* (6-Keto-PGF1*α*, CSB-E14411r) were quantified by ELISA kits (Cusabio Biotech Co., Ltd., Wuhan, China). The OD value of the samples was measured sequentially at 450 nm wavelength with a microplate reader.

### 2.12. Statistical Analysis

SPSS 19.0 statistical software was performed to analyze the experimental data. If normality and homogeneity of variance are met, a *t*-test or one-way ANOVA was used for comparison between the two groups. Multi-factor ANOVA was used for multiple groups. All quantitative data are represented as mean ± standard deviation (SD). *P* < 0.05 indicates the results to be statistically significant.

## 3. Results

### 3.1. TSD Promotes Trabecular Bone Repair in Fractured Rats

**Figure 1:**
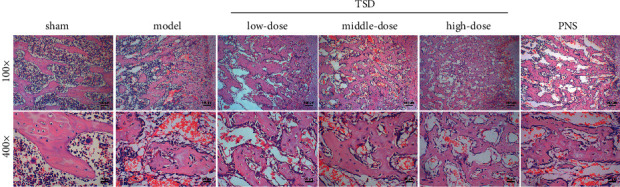
Effects of TSD on trabecular bone repair in fractured rats. After HE staining, the trabecular bone was observed with a 100× and 400× optical microscope; scale bar, 100 *μ*m and 25 *μ*m. The pink area represents the trabecular bone. TSD: Taohong Siwu decoction; PNS: panax notoginsenoside; and HE: hematoxylin-eosin.

The identification and analysis of the components of TSD have been reported in our previous study [19]. We performed HE staining and analysis of the bone tissue in rats treated with TSD 28 days after fracture (Figure 1). The area of the trabecular bone in TSD groups increased compared with the model group. As the dose of TSD increased, the area of trabecular bone increased correspondingly. The trabecular bone thickness was thin in fractured rats, while it was significantly thicker in the low-, medium-, and high-dose TSD groups. Also, the trabecular bone gap was reduced in the low-, medium-, and high-dose TSD groups compared with the control group. The performance of the TSD group was better than that of the PNS group in all cases. The abovementioned results show that TSD can effectively promote trabecular bone repair in fractured rats.

### 3.2. Effects of TSD on Anti-Inflammation and Coagulation

**Figure 2:**
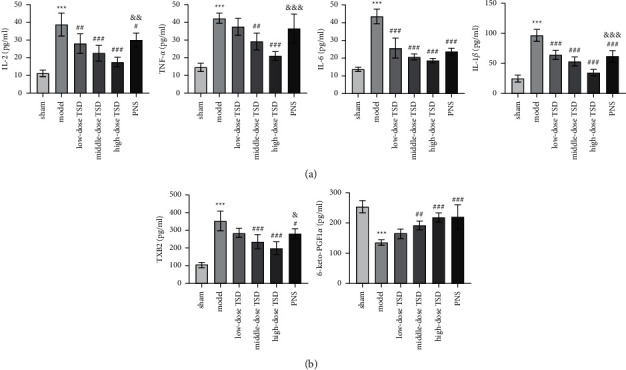
Effects of TSD on anti-inflammation and coagulation. (a) The contents of IL-2, TNF–*α*, IL-6, and IL-1*β*. (b) The contents of TXB2 and 6-keto-PGF1*α*. All indexes were detected by ELISA. ^*∗*^*P* < 0.05, ^*∗∗*^*P* < 0.01, and ^*∗∗∗*^*P* < 0.001 vs. the sham group, ^#^*P* < 0.05, ^##^*P* < 0.01, and ^###^*P* < 0.001 vs. the model group, ^&^*P* < 0.05, ^&&^*P* < 0.01, and ^&&&^*P* < 0.001 vs. the high-dose TSD group. TSD: Taohong Siwu decoction; PNS: panax notoginsenoside; IL-2: interleukin-2; TNF-*α*: tumor necrosis factor-*α*; IL-6: interleukin-6; IL-1*β*: interleukin-1*β*; TXB2: thromboxane B2; 6-keto-PGF1*α*: 6 ketone prostaglandin F1*α*; and ELISA: enzyme-linked immunosorbent assay.

To understand the anti-inflammatory effects of TSD, we measured the levels of inflammatory factors in the plasma of fractured rats by ELISA. IL-2, TNF-*α*, IL-6, and IL-1*β* secreted by the RAECs were obviously reduced under middle- and high-dose TSD treatment compared to the model group (*P* < 0.01). The levels of these inflammatory cytokines were inversely proportional to the dose of TSD. Compared to the positive model group (PNS), TSD groups (especially high dose) showed better inhibitory effects on inflammatory factors (Figure 2(a)). As shown in Figure 2(b), the level of TXB2 in the low-, medium- (*P* < 0.001), and high-dose (*P* < 0.001)TSD serum groups was lower than that in the model group, and the decreasing trend was more pronounced with increasing TSD dose (Figure 2(b)). We noted that the inhibitory effect of TSD at the high dose was better than that of PNS (*P* < 0.05). In comparison, the concentration of 6-keto-PGF1*α* showed the opposite trend. Since high-dose TSD had the best efficacy, the next experiments were conducted using high-dose TSD.

### 3.3. TSD Promotes the Angiogenesis and Migration of the RAECs

**Figure 3:**
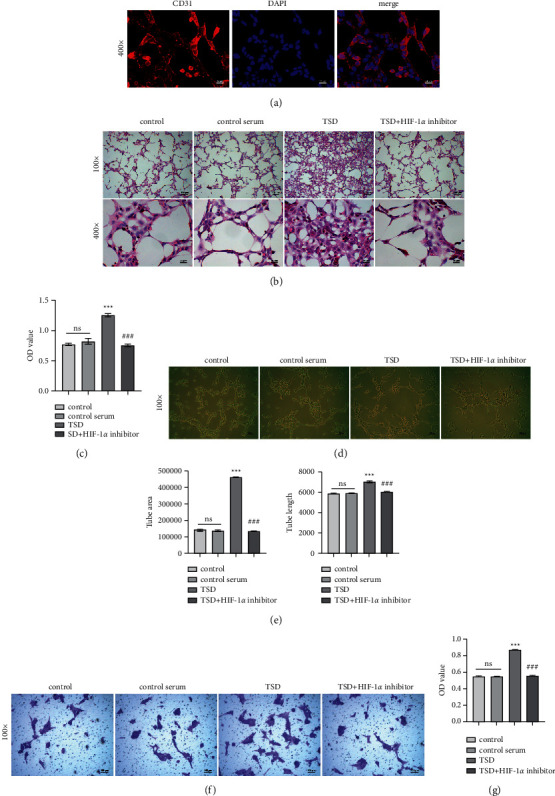
TSD promotes the proliferation, angiogenesis, and migration of the RAECs. (a) Immunofluorescence analysis for primary RAECs identification (400×); scale bar, 25 *μ*m. (b) Representative images and quantification statistics of HE-stained RAECs (100× and 400×); scale bar, 100 *μ*m and 25 *μ*m. (c) MTT detection of RAECs proliferation. (d) Representative images of tube forming experiment (100×); scale bar, 100 *μ*m. (e) Quantitative statistics of (*D*). (f) Representative images of transwell migration experiment (100×); scale bar, 100 *μ*m. (g) The OD value of migrated cells from the migration experiment. ns: no statistical significance; ^*∗∗∗*^*P* < 0.001 vs. the control serum group. ^###^*P* < 0.001 vs. the TSD group. TSD: Taohong Siwu decoction; RAECs: rat aortic endothelial cells; HE: hematoxylin-eosin; MTT: methyl tetrazolium; and OD: optical density.

To understand the effects of TSD on angiogenesis and migration of the RAECs, we first isolated and obtained primary RAECs. CD31 is a marker of the RAECs [27]. To identify whether the extraction of the RAECs was successful, we performed immunofluorescence and HE staining experiments. The results showed that the RAECs were polygonal. CD31 was expressed in the RAECs, suggesting that the extraction was successful (Figures 3(a) and 3(b)). Cell proliferation after 24 h of TSD-containing serum intervention was dramatically better than in the other groups. The number of cells was significantly increased in the TSD group compared with the control serum group (*P* < 0.001). But, the cells in the TSD + HIF-1*α* inhibitor group were fewer than in the TSD group (Figures 3(b) and 3(c), *P* < 0.001). By matrigel tubulogenesis assay, compared with the control serum group, the tube area and length of tube formations were significantly increased in the TSD group (*P* < 0.001). However, the HIF-1*α* inhibitor reversed the changes in tube formation of TSD on the RAECs (Figures 3(d) and 3(e), *P* < 0.001). Cell migration assay showed that TSD-containing serum treatment significantly enhanced the migration of the RAECs compared with the control serum group (*P* < 0.001). The migration of the RAECs was strongly inhibited in the TSD + HIF-1*α* inhibitor group compared to the TSD group (Figures 3(f) and 3(g), *P* < 0.001). Hence, we hypothesized that the functional regulation of the RAECs by TSD might be related to the HIF-1*α* signaling pathway.

### 3.4. TSD Promotes Angiogenesis by Regulating the HIF-1*α* Signaling Pathway

**Figure 4:**
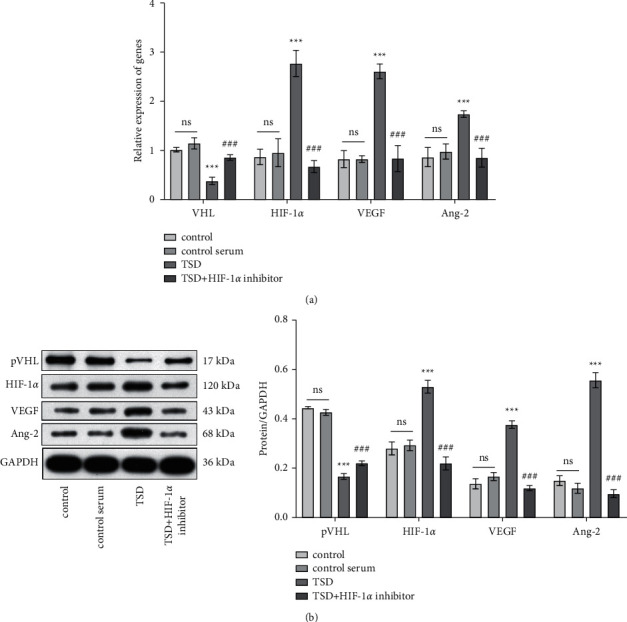
TSD promotes angiogenesis by regulating the HIF-1*α* signaling pathway. (a) Quantitative analysis for the mRNA level of VHL, HIF-1*α*, VEGF, and Ang-2. (b) The protein expression levels of pVHL, HIF-1*α*, VEGF, and Ang-2 were analyzed by western blot. ns: no statistical significance, ^*∗∗∗*^*P* < 0.001 vs. the control serum group. ^###^*P* < 0.001 vs. the TSD group. TSD: Taohong Siwu decoction; HIF-1*α*: hypoxia-inducible factor-1*α*; VHL: von Hippel-Lindau tumor suppressor; VEGF: vascular endothelial growth factor; and Ang-2: angiopoietin-2.

To inquire whether TSD acts on the RAECs through the HIF-1*α* signaling pathway, we examined the expression of HIF-1*α* downstream genes accordingly. At the mRNA level, TSD-containing serum upregulated the expression of HIF-1*α*, VEGF, and angiopoietin-2 (Ang-2) and downregulated von Hippel-Lindau tumor suppressor (VHL) in the RAECs (*P* < 0.001), whereas HIF-1*α* inhibitor greatly reversed the effects of the TSD serum (Figure 4(a), *P* < 0.001). As seen in Figure 4(b), compared with the control serum group, the expression of HIF-1*α*, VEGF, and Ang-2 was significantly increased at the protein level in the TSD serum group (*P* < 0.001). Compared to the TSD serum group, these protein expression levels were significantly inhibited in the TSD + HIF-1*α* inhibitor group (*P* < 0.001). In contrast, pVHL expression was suppressed in the TSD group (*P* < 0.001). HIF-1*α* inhibitor reversed the suppression of pVHL by TSD (*P* < 0.001).

### 3.5. TSD Promotes the Expression Levels of Osteogenic Protein

**Figure 5:**
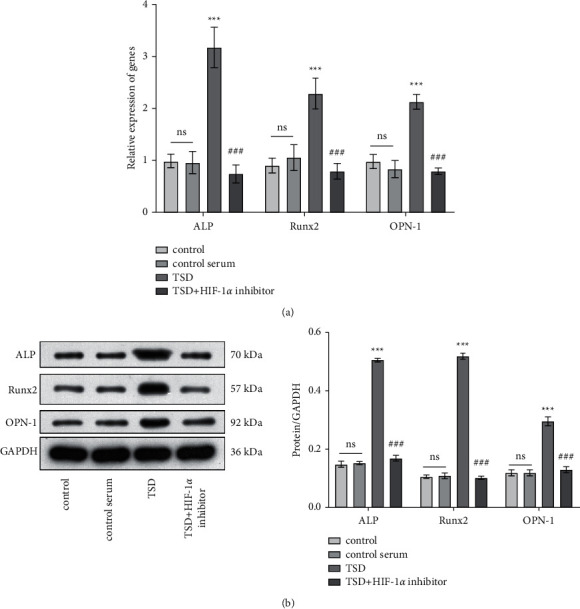
TSD promotes the expression levels of osteogenic protein. (a) Quantitative analysis of ALP, Runx2, and OPN-1 mRNA level. (b) The protein expression levels of quantitative analysis ALP, Runx2, and OPN-1 were analyzed by western blot. ns: no statistical significance; ^*∗∗∗*^*P* < 0.001 vs. the control serum group. ^###^*P* < 0.001 vs. the TSD group. TSD: Taohong Siwu decoction; ALP: alkaline phosphatase; Runx2: runt-related transcription factor 2; and OPN-1: osteopontin-1.

qRT-PCR results demonstrated that the expression of alkaline phosphatase (ALP), runt-related transcription factor 2 (Runx2), and osteopontin-1 (OPN-1) were significantly upregulated in the TSD group compared with the control serum group (Figure 5(a), *P* < 0.001). However, the expression of these genes was downregulated in the TSD + HIF-1*α* inhibitor group compared to the TSD group (*P* < 0.001). The expression of these genes at the protein level was consistent with the mRNA level (Figure 5(b)).

## 4. Discussion

It is known that angiogenesis is a necessary condition for bone repair and regeneration after fracture [16]. When a fracture occurs, the body releases a variety of cytokines such as VEGF, FGF, Ang-1, and Ang-2. These factors jointly promote angiogenesis at the fracture end and accelerate fracture healing [28]. Among these, VEGF is the most important angiogenic factor in the body. Many findings showed that VEGF is one of the main mechanisms for the tight integration of angiogenesis and osteogenesis during fracture repair [29, 30]. Angiopoietin has been reported as another important regulatory factor of angiogenesis besides VEGF. Ang-2 can increase the capillary diameter, reconstruct the basement membrane, and promote EC proliferation and migration under the synergy of VEGF so as to stimulate angiogenesis [31]. In our study, under TSD treatment, the accumulation of HIF-1*α* in the upstream HIF-1*α* pathway effectively promoted the expression of VEGF in the RAECs, so the expression of Ang-2 was correspondingly upregulated. TSD displayed a vital catalytic role in the regeneration of blood vessels at the fracture site. Certainly, the promotion of TSD has also been verified by the morphological observation.

pVHL is an inhibitor of HIF-1 which promotes the degradation of HIF-1*α* protein by the proteasome [32, 33]. We noticed that the expression of pVHL was reduced under the TSD serum group. We proposed that TSD inhibits the production of pVHL, thereby increasing the expression of VEGF and Ang-2 located downstream of HIF-1*α* and ultimately promoting angiogenesis.

So far, increasing evidence has demonstrated the important role of Runx2 in regulating osteogenic differentiation [34]. As a component of the Runt family, Runx2 plays an important part in the transcription of many genes related to osteogenic differentiation [35, 36]. Runx2 can induce the synthesis of collagen type I (COL I), osteocalcin (OC), and OPN, as well as the maturation of osteoblast phenotype [37]. OPN is a multifunctional extracellular matrix (ECM) protein that has been shown to play a regulatory role in angiogenesis and osteoclasts in bone reconstruction [38]. Here, we examined the expression of osteogenesis-related genes, Runx2, ALP, and OPN-1 at the protein and RNA levels. TSD promotes an increase in trabecular area ratio and densities in fractured rats, which may be caused by the increased expression of Runx2, ALP, and OPN-1. These also reflect the effective promoting effect of TSD on osteogenesis in fracture healing.

A bone fracture can cause a high degree of inflammation in the immune system, typically characterized by increased secretion of pro-inflammatory cytokines [39]. IL-6, a member of the pro-inflammatory cytokine family, has been confirmed to induce the expression of various acute inflammatory-related proteins and plays a significant role in the proliferation and differentiation of human cells [40]. Furthermore, it is well established that TNF-*α* regulates intraosseous balance by stimulating osteoclastogenesis and inhibiting osteoblast function and can also induce osteoblast differentiation [41]. Previous research has proved that TSD could reduce inflammation by decreasing the levels of TNF-*α* and IL-1*β* in the serum [42]. The present study also confirmed that the plasma levels of IL-2, IL-6, TNF-*α*, and IL-1*β* were decreased in TSD-treated fractured rats. It means that TSD can effectively alleviate the inflammatory reaction caused by the fracture. Based on the obtained results, it was inferred that the alleviative effect of TSD on the inflammatory response caused by fracture enhanced with the increased dose accordingly, within a certain dose range.

TXA2 is a strong vasoconstrictor, which can effectively induce platelet aggregation in the body [43]. Prostacyclin-2 has the effect of inhibiting platelet aggregation and relaxing peripheral blood vessels [44]. Both of them are major indexes to reflect the antiplatelet activity. Considering the difficulty of detection, we reflected the concentration levels by detecting their metabolites TXB2 and 6-keto-PGF1. The data displayed that TSD could decrease the content of TXB2 and increase the content of 6-keto-PGF1 in the rat plasma. The data from our present study demonstrated that TSD had a good antiplatelet activation effect.

## 5. Conclusion

Our study provides direct evidence that TSD promotes early angiogenesis in fractures. TSD-induced upregulation of HIF-1*α* pathway angiogenic protein expression, which promoted early angiogenesis in fractures. In addition, TSD suppressed the overexpression of inflammatory cytokines and platelet activation markers, thereby alleviating fracture-induced inflammation and accelerating wound healing. In summary, TSD may be a potential fracture healing agent by regulating the HIF-1*α* pathway.

## Data Availability

The data used to support the findings of this study are available from the corresponding author upon request.

## Supplementary Materials

Supplementary Materials Supplementary Table 1: primers for qRT-PCR. Supplementary Table 2: antibody information.
