# Supplementary material for: Taohong Siwu Decoction Promotes Osteo-Angiogenesis in Fractures by Regulating the HIF-1α Signaling Pathway
Source: Evid Based Complement Alternat Med. 2022 Sep 20;2022:6777447. doi: 10.1155/2022/6777447 (PMC9526655; doi:10.1155/2022/6777447)
Supplement: Supplementary Materials — Supplementary Table 1: primers for qRT-PCR. Supplementary Table 2: antibody information. [file 6777447.f1.zip › 6777447.f1/Supplementary Table 1.docx]

| **Supplementary Table 1. Primers for qRT-PCR** | | |
| --- | --- | --- |
|  | Forward | Reverse |
| HIF-1α | ACGATTGTGAAGTTAATGCTCCC | AACCAACAGAAACGAAACCCC |
| VEGF | CCTCGTCCTCTCCCTACCCCACT | TCCTGCCCCATTGCTCTGTACCTT |
| ALP | TGTGAGGCCGGTTACTAAAGTCCA | ACACACAAAGCACTCGGGGT |
| RUNX2 | CCACCCAGTAGCAAACCGAA | GCATCAGACAAACACACGGAC |
| OPN-1 | TACCAGAAAAGAACCGCGAGA | TGCCAAAGTGTTCAATCAGGA |
| VHL | TGCCAACATCACATTGCCAGT | CTTCATAGAGCGACCTGACGAT |
| Ang-2 | ATGTGGCAGATTGTTTTCCTAACTT | CGTTCTGGACCTGGTACTGC |
| GAPDH | ACAGCAACAGGGTGGTGGAC | TTTGAGGGTGCAGCGAACTT |

HIF-1α: hypoxia-inducible factor-1α; VEGF: vascular endothelial growth factor; ALP: alkaline phosphatase; Runx2: runt-related transcription factor 2; OPN-1: Osteopontin-1; VHL: von Hippel-Lindau tumor suppressor; Ang-2: Angiopoietin-2.
