# Supplementary material for: Taohong Siwu Decoction Promotes Osteo-Angiogenesis in Fractures by Regulating the HIF-1α Signaling Pathway
Source: Evid Based Complement Alternat Med. 2022 Sep 20;2022:6777447. doi: 10.1155/2022/6777447 (PMC9526655; doi:10.1155/2022/6777447)
Supplement: Supplementary Materials — Supplementary Table 1: primers for qRT-PCR. Supplementary Table 2: antibody information. [file 6777447.f1.zip › 6777447.f1/Supplementary Table 2.docx]

| **Supplementary Table 2. Antibody information** | | | | |
| --- | --- | --- | --- | --- |
|  | Catalog No. | Dilution Ratio | Company | Contry |
| pVHL | ab77262 | 1:2000 | Abcam | UK |
| HIF-1α | 20960-1-AP | 1:2000 | Proteintech | USA |
| VEGF | 66828-1-Ig | 1:1000 | Proteintech | USA |
| Ang-2 | 24613-1-AP | 1:800 | Proteintech | USA |
| ALP | ab133602 | 1:30000 | Abcam | UK |
| Runx2 | ab76956 | 1:1000 | Abcam | UK |
| OPN-1 | 11076-1-AP | 1:500 | Proteintech | USA |
| GAPDH | 10494-1-AP | 1:4000 | Proteintech | USA |
| HRP goat anti-mouse IgG | SA00001-1 | 1:2000 | Proteintech | USA |
| HRP goat anti-rabbit IgG | SA00001-2 | 1:2000 | Proteintech | USA |
| HRP donkey anti-goat IgG | SA00001-3 | 1:1000 | Proteintech | USA |

pVHL: phosphorylation of von Hippel-Lindau tumor suppressor; HIF-1α: hypoxia-inducible factor-1α; VEGF: vascular endothelial growth factor; Ang-2: Angiopoietin-2; ALP: alkaline phosphatase; Runx2: runt-related transcription factor 2; OPN-1: Osteopontin-1.
